# Intraoperative Assessment of Parathyroid Viability using Laser Speckle Contrast Imaging

**DOI:** 10.1038/s41598-017-14941-5

**Published:** 2017-11-01

**Authors:** E. A. Mannoh, G. Thomas, C. C. Solórzano, A. Mahadevan-Jansen

**Affiliations:** 1 Department of Biomedical Engineering, Vanderbilt University, Nashville, TN 37235 USA; 2 Division of Surgical Oncology and Endocrine Surgery, Vanderbilt University Medical Center, Nashville, TN 37232 USA; 3 Vanderbilt Biophotonics Center, Vanderbilt University, Nashville, TN 37235 USA

## Abstract

Post-surgical hypoparathyroidism and hypocalcemia are known to occur after nearly 50% of all thyroid surgeries as a result of accidental disruption of blood supply to healthy parathyroid glands, which are responsible for regulating calcium. However, there are currently no clinical methods for accurately identifying compromised glands and the surgeon relies on visual assessment alone to determine if any gland(s) should be excised and auto-transplanted. Here, we present Laser Speckle Contrast Imaging (LSCI) for real-time assessment of parathyroid viability. Taking an experienced surgeon’s visual assessment as the gold standard, LSCI can be used to distinguish between well vascularized (n = 32) and compromised (n = 27) parathyroid glands during thyroid surgery with an accuracy of 91.5%. Ability to detect vascular compromise with LSCI was validated in parathyroidectomies. Results showed that this technique is able to detect parathyroid gland devascularization before it is visually apparent to the surgeon. Measurements can be performed in real-time and without the need to turn off operating room lights. LSCI shows promise as a real-time, contrast-free, objective method for helping reduce hypoparathyroidism after thyroid surgery.

## Introduction

Approximately 8 million Americans are diagnosed annually with some form of thyroid disease^1^, over 92,000 of whom undergo partial or total thyroidectomy as definitive treatment^2^. An estimated 3% of these procedures result in the patient being permanently unable to produce sufficient levels of parathyroid hormone for normal serum calcium regulation^3,4^. Additionally, a significant proportion of patients suffer from a transient form of this post-surgical hypoparathyroidism and consequent hypocalcemia^5–8^, with some studies reporting incidences as high as 47%^8^. Hypocalcemia can lead to cardiac arrhythmias, muscle spasms, tetany and eventually death, and is a huge economic burden for these patients who may require extended hospital stays or have to take regular calcium supplements for the rest of their lives to prevent these effects^9^. It is also one of the main causes for malpractice lawsuits after endocrine surgery^7^. Post-surgical hypoparathyroidism results from accidental disruption of the blood supply to, or accidental removal of, otherwise healthy parathyroid glands, organs responsible for regulating calcium. There are typically four parathyroid glands, which contain calcium-sensing receptors and secrete parathyroid hormone (PTH) in response to low serum calcium. Parathyroid hormone acts on bone to promote resorption, on the kidneys to reduce calcium elimination in urine, and on the intestines to promote calcium absorption through the intestinal wall, all of which help to increase serum calcium^10^. Vascular compromise of parathyroid glands may occur after thyroidectomy because of the close proximity of the parathyroid glands to the thyroid. In many cases, the parathyroid glands derive their blood supply from the same blood vessels that feed the thyroid^11^. Fortunately, devascularized parathyroid glands can be salvaged by autotransplantation – a procedure by which the gland is excised and transplanted typically into the sternocleidomastoid muscle^12^. However, identifying these glands is challenging and currently depends on subjective visual assessment and surgeon experience. Additionally, failure to revive parathyroid function after autotransplantation has been reported to occur in 14–17% of cases^12,13^. As a result, a surgeon needs to be certain that a parathyroid gland is devascularized, and therefore not viable if left in place, before committing to this procedure.

A number of techniques have been employed to assess parathyroid viability intraoperatively. A common approach is to look for bright red bleeding after pricking the parathyroid gland with a needle or cutting off tiny fragments^14^, however care needs to be taken not to irreversibly damage the gland. Topical application of a dilute lidocaine solution has been employed to cause vasodilation in vascularized parathyroid glands. Any gland that does not swell after application of lidocaine is considered not viable and is therefore autotransplanted. This procedure is risky, since lidocaine can cause paralysis of the vocal cords if it comes into contact with the exposed laryngeal nerve^14^. Intraoperative measurement of PTH is performed routinely in parathyroidectomies to confirm removal of the hyperactive gland. Since the half-life of PTH in the blood is 3–5 minutes, removal of a parathyroid gland leads to a noticeable decrease in serum PTH within a relatively short amount of time. Based on this concept, intraoperative measurement of PTH has also been employed in thyroidectomies^15^, however this approach gives no indication of which parathyroid gland might be compromised. Another approach is to use indocyanine green (ICG) angiography to identify devascularized parathyroid glands. One study reported that hypoparathyroidism did not occur after surgery in thyroidectomy patients where at least one parathyroid gland was determined (by qualitative assessment of ICG fluorescence) to be well vascularized^16^. This method requires administration of an exogenous contrast agent and is limited by how frequently it can be performed. There is therefore a critical need for a real-time, contrast-free and objective method for assessing the viability of parathyroid glands intraoperatively.

Here, we present the first report on the application of Laser Speckle Contrast Imaging (LSCI) to meet this need. This technique was first introduced in the 1980′s as a photographic method for obtaining spatial maps of the velocity distribution in a field^17^. With the advancement of digital technology, it was later developed into a digital technique which made its application more streamlined^18^. The technique analyzes the interference pattern produced when coherent light is incident on a surface. Minute differences in path length created by the light waves scattering from different regions produce bright and dark spots of constructive and destructive interference respectively, termed as a speckle pattern. This speckle pattern fluctuates depending on how fast particles are moving within a few hundred microns of the surface. Blurring of the speckle pattern occurs when the motion is fast relative to the integration time of the detector. Analyzing this spatial blurring provides contrast between regions of faster versus slower motion and forms the basis of LSCI^19^. Quantitatively, the speckle contrast is defined as the ratio of the standard deviation of pixel values to the mean (Equation 1), calculated within a given region.

1 $${K}_{s}=\frac{{\sigma }_{s}}{\langle I\rangle }$$

This technique is sensitive to microvascular blood flow and has been employed in a variety of tissues where the vessels of interest are generally superficial, such as the retina, skin and brain^19^. Many of its applications have been in laboratory settings for investigating phenomena such as skin blood flow dynamics in response to external stimuli^20,21^, and cerebral blood flow in animal models of stroke^22^. Clinically, LSCI and similar techniques have been applied to monitoring Port Wine Stain laser therapy^23^, correlating microvascular blood flow with healing time in burn wounds^24^, and measuring cerebral blood flow in patients undergoing brain tumor resection^25^. However, there are no published reports on the application of LSCI or any other label-free optical method for assessing parathyroid gland viability during endocrine surgery. Parathyroid glands are densely packed with blood vessels, given that they secrete PTH to the entire body. Furthermore, their small size (3–8 mm)^11^ makes many of these vessels superficial, making these glands suitable targets for assessment using LSCI.

The goal of this study is to evaluate the capability of LSCI to distinguish between vascularized and compromised parathyroid glands as determined by an experienced surgeon, in real-time during endocrine surgery. This pilot study was performed in patients undergoing thyroidectomies and parathyroidectomies by measuring the speckle contrast of parathyroid glands *in vivo*. Performance of LSCI was evaluated in thyroidectomies, by grouping speckle contrast from normal functioning and compromised parathyroid glands according to the surgeon’s classification. Validation was performed in parathyroidectomies, where the state of vascularity of the parathyroid gland was controlled in glands planned for excision. Results demonstrate the effectiveness of LSCI for parathyroid gland assessment.

## Results

### Device design

**Figure 1:**
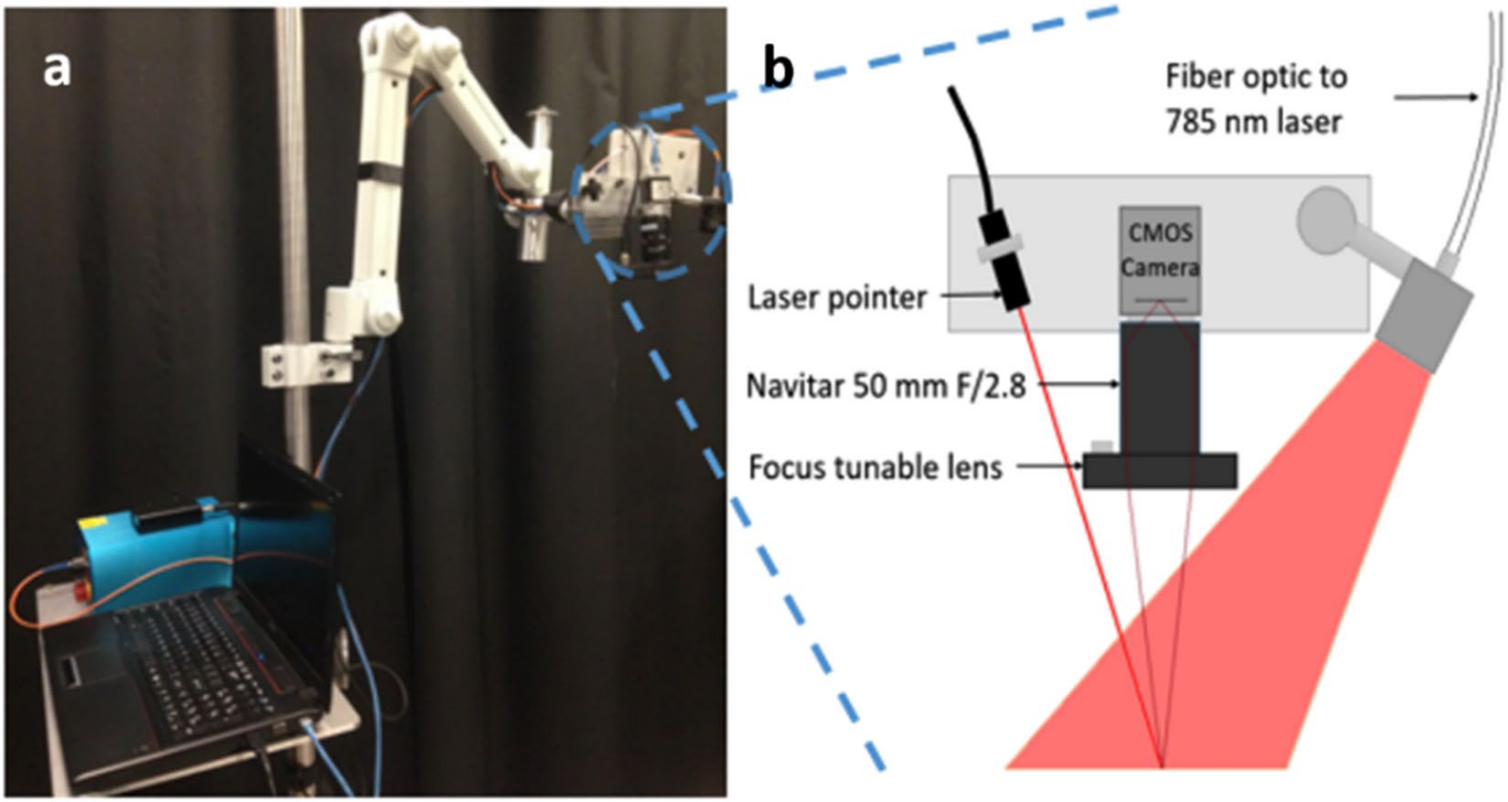
Clinical LSCI system. Picture of device (**a**), schematic of imaging end (**b**).

While commercial LSCI systems exist, a system was developed in-house in order to have full control over the parameter space and make it customizable for the specific application. This system is shown in Fig. 1. In essence, the device consists of a 785 nm coherent light source to diffuse light onto the surgical field, a near-infrared optimized camera to record the speckle pattern produced, and a computer to process and display images at 10 frames per second. (See methods for additional details). The field of view was measured to be about 5 cm × 6 cm at a distance of 45 cm above the operating table and the laser intensity at this distance was 0.6 mW/cm^2^.

### Effect of ambient lighting on speckle contrast images

**Figure 2:**
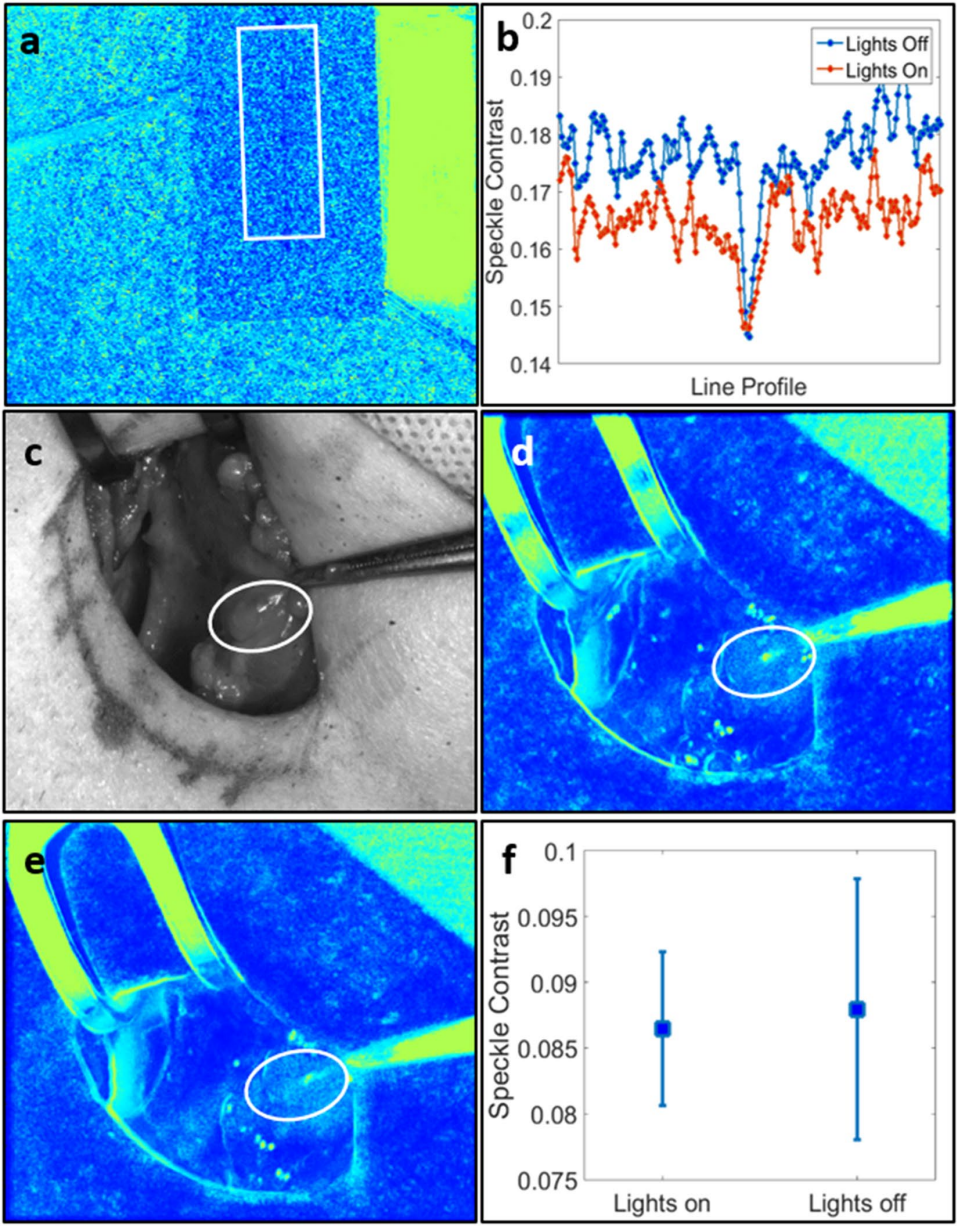
Overhead lights have minimal effect on LSCI system. Figure shows speckle contrast image of microfluidic flow phantom (**a**) and average line profile perpendicular to flow channel for both lighting conditions (**b**); white light image of surgical field with OR lights on (**c**); speckle contrast image with OR lights on (**d**); speckle contrast image with OR lights off (**e**); mean and standard deviation of speckle contrast within parathyroid for both cases (**f**). Parathyroid glands are indicated with white ellipses and the flow phantom region of interest is indicated with a white rectangle.

Performing measurements without the need to turn off the operating room (OR) lights is essential so that any disruption to the surgical workflow is minimized. The effect of ambient lights on speckle contrast was evaluated in the laboratory on a microfluidic flow phantom with a milk powder solution flowing through its 400 µm channel (Fig. 2a,b). Speckle contrast is displayed as false-colour images throughout this manuscript to distinguish them from white light images which are in grayscale. A rectangular region of interest crossing the channel was selected and line profiles perpendicular to the channel within this region were averaged for both conditions, lights on and lights off. The average speckle contrast across the surface of the phantom dropped by 0.01 when the lights were turned on, however remained about the same within the channel.

To determine how room lights would affect LSCI data acquired in the operating room, the system was tested *in vivo* on a patient undergoing parathyroidectomy. One set of images was acquired with the room lights on and the surgical lights above the operating table pointed away from the surgical field. (The lights above the operating table had to be turned away due to their much higher intensity which saturated the camera and did not permit acquisition of useful LSCI data). Another set of images was then acquired with all lights off. For each condition, 20 speckle contrast images were averaged after acquisition and the same region of interest was used to calculate average parathyroid gland speckle contrast. There was a 1.7% decrease in the average speckle contrast value of the parathyroid gland when the OR lights were left on, compared to when they were off (Fig. 2c–f). These decreases in speckle contrast are to be expected since room/OR lights have a broad frequency spectrum^26^ and therefore do not produce a speckle pattern. The uniform lighting fills in dark spots in the speckle pattern generated by the laser, thereby reducing the contrast. However, the magnitude of this decrease in the case of the *in vivo* parathyroid gland measurement (~0.002) was miniscule compared to the possible range of speckle contrast values (0 to 1). Additionally, this value is less than 2% of the range of average parathyroid gland speckle contrast obtained in this study. These results indicated that the performance of LSCI is negligibly affected by ambient light and validates its application in the operating room with the OR lights on but pointed away from the patient during image acquisition.

### Vascularized vs. compromised parathyroid glands

**Figure 3:**
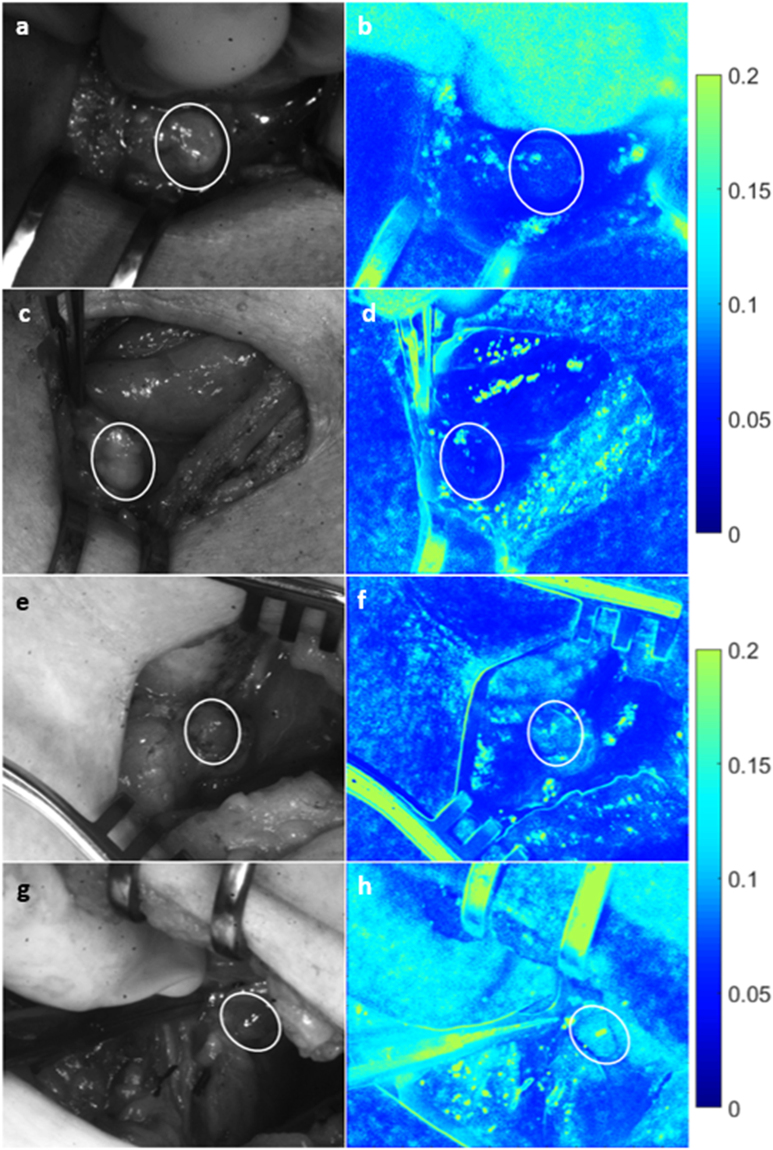
Speckle contrast is lower for well vascularized parathyroid glands. Images are shown for two examples each of vascularized (**a**–**d**) and compromised (**e**–**h**) parathyroid glands. Left column (**a**,**c**,**e**,**g**) shows white light images, with the corresponding speckle contrast images (**b**,**d**,**f**,**h**) in the right column. Parathyroid glands are indicated with white ellipses.

The LSCI system was tested in twenty patients undergoing thyroidectomy at Vanderbilt University Medical Center. Speckle contrast images of parathyroid glands were acquired during the course of surgery and at the discretion of the participating surgeon. The surgeon’s visual assessment of the gland’s viability (without input from speckle contrast images) was recorded at the time of image acquisition for each gland. Figure 3 shows examples of parathyroid glands considered vascularized and compromised. The left column shows white light images of the surgical field, and the right column shows the corresponding speckle contrast image (average of 20 images). Parathyroid glands are indicated with a white ellipse. As these images show, parathyroid glands considered vascularized by the surgeon have lower speckle contrast than those considered to be compromised.

**Figure 4:**
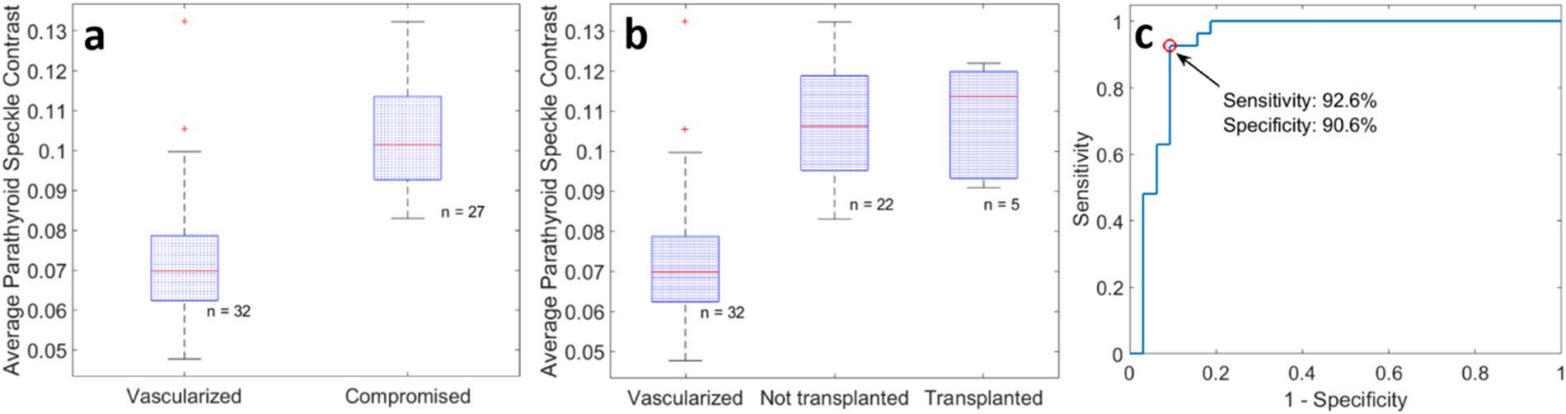
Average parathyroid gland speckle contrast grouped according to surgeon’s assessment. Speckle contrast is significantly lower (p < 0.0001) for vascularized glands (**a**). Of the compromised glands only 5 required transplantation, however no significant difference was observed between this sub-group and the compromised glands that were left in place (**b**). Classifying parathyroid glands based on average speckle contrast generated an ROC curve with an area under the curve of 0.935, and an optimum point with sensitivity and specificity of 92.6% and 90.6% respectively (**c**).

The average speckle contrast within each parathyroid gland was calculated, avoiding bright spots of specular reflection. This data was then grouped according to the surgeon’s visual assessment of vascularity, which served as the gold standard. Using a two-sample two-sided Student’s t-test, a statistically significant difference (p < 0.0001) was observed in the speckle contrast between glands considered vascularized versus compromised as determined by the surgeon (Fig. 4a). Within the compromised group, there were 5 glands that were transplanted, and 22 others that the surgeon decided could be left in place without significant adverse effects to the patient. While the mean speckle contrast was higher in the transplanted group, this difference was not found to be significant using a two-sample t-test. These results are shown in Fig. 4b.

Using speckle contrast as a classifier to distinguish between the vascularized and compromised parathyroid glands, a receiver operating characteristic (ROC) curve was generated with an area under the curve of 0.935 (Fig. 4c). The optimum threshold for distinguishing between the two groups was found to be 0.09, which resulted in a sensitivity of 92.6% (25/27 compromised glands correctly identified) and a specificity of 90.6% (29/32 vascularized glands correctly identified). From this, the overall accuracy was calculated to be 91.5%.

### Validation of Technique

Given that assessment of parathyroid gland viability currently relies on a surgeon’s subjectivity, and these normal functioning glands cannot be excised for histological validation without compromising patient care, validation of LSCI for this application was performed on eight patients undergoing parathyroidectomy, where a diseased parathyroid gland is planned for removal. As part of standard procedure, the surgeon ties off all blood supply to the gland prior to excision. This provides a controllable scenario for evaluating the capability of the device to detect when a parathyroid gland is compromised as well as to determine the detection limit (or time) of the system. To confirm removal of the hyperactive parathyroid gland, intraoperative measurement of PTH is routinely performed before and 10 minutes after excision of the gland.

**Figure 5:**
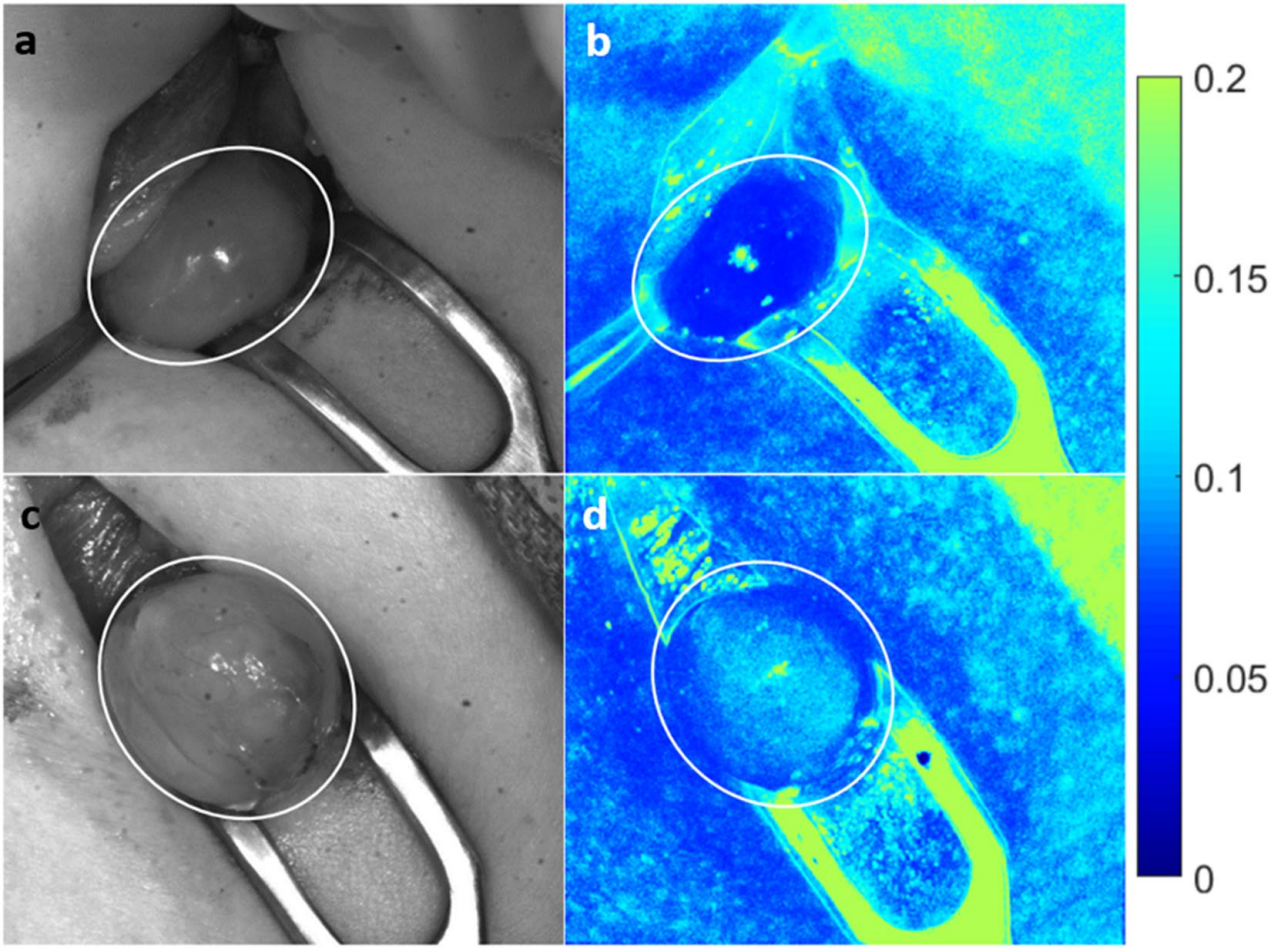
Change in speckle contrast occurs seconds after blood supply ligation. A noticeable change in speckle contrast (**b**,**d**) was observed in the same parathyroid gland seconds after tying off blood supply to the gland, however no such change was observed in white light images (**a**,**c**). Images (**a**) and (**b**) were taken before, while images (**c**) and (**d**) were taken about 30 seconds after ligation of the blood supply. Parathyroid gland is indicated with a white ellipse.

An example of a hypercellular diseased parathyroid gland marked for removal is shown in Fig. 5. Again, the left column shows white light images while the corresponding speckle contrast images are in the right column. Images on the top row are of the gland before the surgeon tied off the blood supply. Images on the bottom row were taken on average 30 seconds after the surgeon tied off the blood supply to the gland. According to the surgeon and as seen in the white light image, this gland did not appear visually different from its initial state. However, there is a noticeable change in the speckle contrast images. In all cases, speckle contrast increased by a minimum of 18% after the surgeon tied off the blood supply (data shown in Table 1; mean increase of 0.043). Further, post-excision intraoperative PTH was also lower than pre-excision PTH, confirming removal of the diseased gland.

**Table 1:** Summary of data from excised diseased parathyroid glands.

Patient #	Pre-ligation speckle contrast	Post-ligation speckle contrast	Pre-excision [PTH] (pg/mL)	Post-excision [PTH] (pg/mL)
1	0.076	0.126	86	26
2	0.057	0.094	142	34
3	0.062	0.131	172	32
4	0.074	0.130	119	115
4	0.069	0.119	115	45
5	0.064	0.093	151	53
5	0.048	0.085	53	N/A
5	0.084	0.113	N/A	N/A
6	0.063	0.092	130	31
7	0.067	0.115	76	26

In all cases there was a large increase in speckle contrast seconds after ligation of the blood supply to the gland. There was also a decrease in PTH related to the number of diseased glands removed. In patient 5, LSCI data was obtained from 3 glands that were excised, however PTH measurement was not performed after removal of glands 2 and 3.

## Discussion

This work demonstrates the capability of LSCI to intraoperatively distinguish between vascularized and compromised parathyroid glands. These measurements can be performed in real-time and with the operating room lights on, minimizing disruption to the surgical workflow. The average speckle contrast value of vascularized parathyroid glands was significantly lower than that of the compromised parathyroid glands, consistent with the understanding that reduced blood flow causes less blurring of the speckle pattern and therefore a higher speckle contrast^19^. Using the ROC curve in Fig. 4c, a speckle contrast value of ~0.09 was found to be optimal in distinguishing between the two groups with 91.5% accuracy. The ability of LSCI to accurately detect compromised vascularity in the parathyroid gland was validated in the parathyroidectomy cases. These images further show that this device is able to detect changes in speckle contrast within seconds of devascularization. This is much earlier than a surgeon would be able to identify based on visual inspection alone which relies on the gland turning dark with deoxygenated blood and losing turgor, a process which can take several minutes and is often missed. Unlike other techniques that have been used to assess parathyroid gland viability, LSCI can be performed non-invasively, with no risk of damage to the parathyroid gland by physical trauma, nor damage to any nearby tissues. It does not require administration of an exogenous contrast agent, which can take 1–2 minutes to achieve optimum circulation^16^. Additionally, multiple measurements can be made on the same gland without risk of toxicity. The device enables real-time assessment by processing and displaying speckle contrast images at 10 frames per second. The images shown in this manuscript were created after acquisition by averaging individual frames in order to improve spatial resolution. However, quantitative information on a region of interest can also be displayed in real-time if desired as this information is generated concurrently with imaging. This technique can be performed with the room lights on, only requiring that the lights above the operating table be pointed away from the surgical field.

The biggest challenge facing this study is the fact that the current gold standard for assessing parathyroid gland viability intraoperatively relies on the surgeon’s experience which is highly subjective. The surgeon that participated in this study has multiple years of experience in this field and is therefore more likely than not to provide accurate assessments. However, thyroidectomies are routinely performed by general surgeons and residents who may perform less than 25 of these cases in a year. According to the American College of Surgeons this is likely to result in a higher error rate^27^, indicating the need for objective assessment of parathyroid viability. These results demonstrate that LSCI has the potential to become the gold standard in this field.

In the absence of an objective gold standard to validate the performance of this device, paired measurements were made in parathyroidectomy cases where the state of vascularity was controlled. It should be noted that the PTH measurements made after excision of the glands are not meant to directly correlate with speckle contrast values but are simply to confirm that the excised tissues are diseased parathyroid glands. A more robust validation method would be to measure PTH after tying off the gland but before excision. However, this was not feasible as it takes about 5–10 minutes for PTH levels to stabilize to a new value and such a procedure would extend OR time and lead to added risk for the patient.

**Figure 6:**
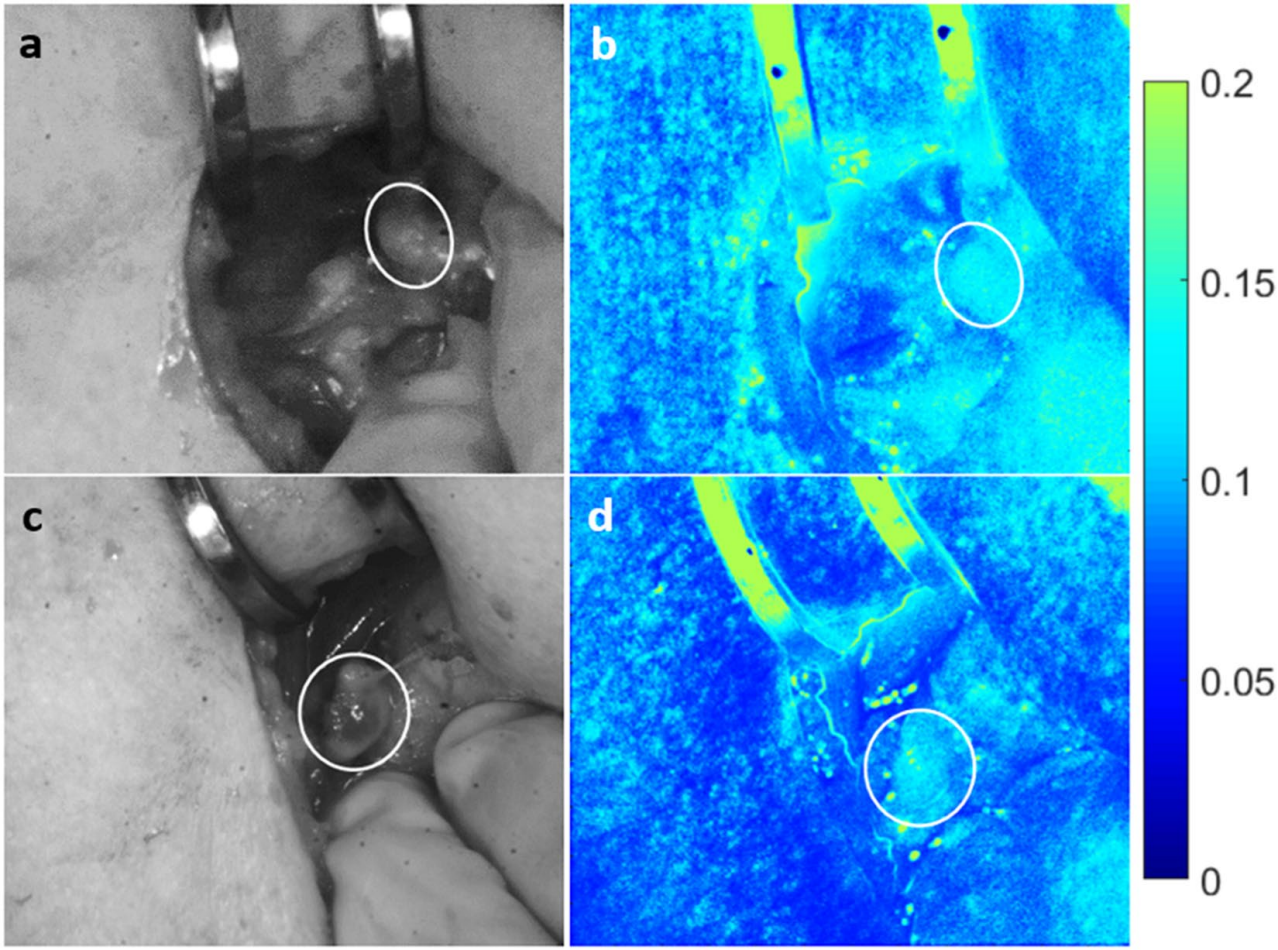
LSCI detects vascular compromise in healthy parathyroid gland before it is visually apparent. White light image of a parathyroid gland initially thought well vascularized (**a**) did not agree with speckle contrast image (**b**). After 15 minutes, this gland was re-evaluated as devascularized (**c**) and transplanted. Speckle contrast image (**d**) supported this assessment. Parathyroid gland is indicated with a white ellipse.

While this device performed with high accuracy in distinguishing between vascularized and compromised parathyroid glands in thyroidectomies, there were 5 instances of disagreement with the surgeon. One case shed light on a possible reason for this disagreement. This particular parathyroid gland was evaluated by the surgeon to be initially well vascularized, however its speckle contrast value suggested otherwise as observed in Fig. 6b. After 15 minutes, the surgeon re-evaluated this gland as devascularized and transplanted it, with speckle contrast images agreeing with this assessment (Fig. 6d). This case further demonstrates the ability of LSCI to detect vascular compromise before it is visually apparent to the surgeon.

An interesting observation was that of the 27 glands the surgeon considered to have suffered vascular compromise, only 5 were autotransplanted. The rest were considered capable of recovery. However, no significant differences in speckle contrast were observed between these two groups (though the small sample size for the transplanted group will make any differences difficult to identify). This warrants further investigation with a larger study to identify what is truly viable. Future studies will additionally acquire pre- and post-surgery PTH measurements in patients with autotransplanted and intact parathyroid glands so that patient outcome may be correlated with speckle contrast images. This will enable answering questions such as: “Should all glands with speckle contrast above a given threshold be autotransplanted in order to avoid hypoparathyroidism?”, and/or, “Is it safe to leave suspicious-looking glands in place if at least one parathyroid has speckle contrast below this threshold?”

It should be noted that LSCI senses motion, therefore these measurements are highly susceptible to random movement of the surgical field caused by the patient’s breathing and movement of the hands holding retractors to expose the surgical field. Image registration was used to align images prior to averaging, however this does not avoid the artificial decrease in speckle contrast introduced to a single frame by such motion. For reference, the average speckle contrast of an excised parathyroid gland placed on a stable surface in the same operating room was measured to be 0.2, whereas the highest value obtained *in vivo* was about 0.13. In spite of this unavoidable source of error, very distinct differences in speckle contrast were obtained as demonstrated by the parathyroidectomy data (Table 1). To improve the accuracy of the final image, any image with jerky motion or a headlamp accidentally turned towards the surgical field (visually identified as a global sharp decrease in speckle contrast) was excluded from the averaging. This was done manually due to the rarity of the event. Only a few data points (<5) in the boxplots in Fig. 4 and none of the displayed images suffered from this problem. However, in each case, sufficient data was acquired so that 20 images could still be averaged. In the future, should it be required, an algorithm could be employed to automate this process and reject speckle contrast images with values a specified percentage below the mean. Another point to consider is that while speckle contrast values theoretically range between 0 and 1, in practice this is difficult to achieve. A layer of stationary scatterers above the moving scatterers of interest (for example, epithelial cells making up the walls of blood vessels) introduce a static component to the speckle pattern that makes the minimum obtainable speckle contrast greater than 0. Additionally, spatial averaging of light across individual pixels in the instrument causes the maximum obtainable speckle contrast to be less than 1. This can be corrected for with a linear factor^28^, which could be implemented in future to further improve the present system. Regardless of these limitations, this study was able to demonstrate the applicability of LSCI as a real-time, contrast-free and objective guidance tool for assessing parathyroid gland viability intraoperatively. It will be of particular benefit to less experienced surgeons and residents, and could reduce hospitalization and long-term medication costs associated with post-surgical hypoparathyroidism. This device could also have applicability for other clinical procedures where there is the need to non-invasively investigate the presence of tissue blood flow.

## Conclusion

We present LSCI as a potential real-time, non-invasive, contrast-free and objective tool for guiding surgeons during thyroid surgeries to assess the viability of parathyroid glands. The device developed in this study can provide this information to surgeons with minimal disruption to the surgical workflow, and can detect vascular compromise in its early stages before it becomes evident to the surgeon’s eye. This tool can therefore significantly minimize post-surgical hypoparathyroidism and its consequent morbidities and costs.

## Materials and Methods

### LSCI system design

The LSCI system developed for this study is shown in Fig. 1. It is mounted on an articulated arm attached to a mobile cart. A 785 nm diode laser (Innovative Photonics Solutions, Monmouth Junction, NJ) is coupled through a 400 µm fiber optic patch cord (Thorlabs, Newton, NJ) to a lens tube containing a 75 mm focal length biconvex lens (Thorlabs, Newton, NJ). This lens diverges the laser light to a ~8 cm diameter spot at a distance of about 45 cm from the lens. The irradiance at the surface was measured to be ~0.6 mW/cm^2^. A near-infrared optimized camera (acA1300-60 gmNIR, Basler AG, Ahrensburg, Germany) captures the images, which are focused onto the camera sensor by an imaging lens system (Navitar 50 mm F/2.8, Navitar, Woburn, MA). The camera is aligned vertically, and the angle between the illumination beam and the line of sight of the camera is fixed at approximately 9° and was designed to reduce specular reflection. Attached to the front end of the lens system is a focus tunable lens (EL-16-40-TC-VIS-5D-M27, Optotune, Dietikon, Switzerland). This allows the images to be focused during each procedure from outside the sterile field as the height of the operating table may be slightly adjusted during surgery. The field of view of the camera was measured to be about 5 cm × 6 cm. A 5 mW 660 nm laser pointer (DigiKey, Thief River Falls, MN) is attached such that its beam is co-localized with the center of the camera’s field of view at a distance of 45 cm. The purpose of this laser pointer is to guide the surgeon in positioning the system above a parathyroid gland so that it is approximately in the center of the field of view. Images recorded by the camera are sent to a laptop computer for processing and display.

### Ensuring adequate sampling of speckle pattern

An important consideration when performing LSCI is that the speckle pattern be adequately sampled. The smallest speckle should be at least twice the size of the sensor pixel in order to meet the Nyquist sampling criterion and avoid underestimating speckle contrast^29^. To test whether the developed LSCI system met this criterion, an optical tissue phantom made of polydimethylsiloxane (PDMS) with titanium dioxide to simulate tissue scattering (reduced scattering coefficient of 8 cm^−1^) was illuminated and imaged with the device using different aperture sizes. The power spectrum was analyzed to ensure there was no aliasing. The iris size was set close to f/16 and locked in this position for the entire study. A well-defined energy band centered at the origin of the power spectrum confirmed no aliasing. Although larger apertures also had no aliasing, this aperture size was chosen because it also resulted in very little background from ambient lighting.

### Patient recruitment and imaging protocol

This study was conducted in accordance with the Declaration of Helsinki and its amendments. The study was approved by the Vanderbilt University Medical Center (VUMC) Institutional Review Board (IRB). Patients undergoing partial or total thyroidectomy at the Vanderbilt University Medical Center were recruited and written informed consent was obtained from each patient (n = 20) prior to participation.

Images of parathyroid glands were recorded during the course of surgery at the discretion of the surgeon. The surgeon first determined the state of vascularity of the gland based on visual inspection, and then positioned the device above the surgical field so that the laser pointer beam was on the parathyroid gland. With the camera integration time set to about 50 ms to allow imaging with the room lights on, white light images were initially acquired to identify the location of the parathyroid gland. The laser pointer was turned off and the integration time was then set to 5 ms for the rest of the image acquisitions. All speckle contrast images were generated with an integration time of 5 ms. This integration time is within the range typically used for LSCI^19^, and results in the room lights contributing very little signal to the image while effectively detecting the 785 nm speckle pattern. Next, the 785 nm laser was turned on and images were acquired and sent to the computer for real-time processing of speckle contrast images. Roughly fifteen seconds of acquisition was allowed for each gland to ensure that a sufficient number of images was obtained. The surgeon was blinded to all images during the surgery.

### Validation of technique

Eight patients undergoing parathyroidectomy at the VUMC were recruited and written informed consent obtained prior to participation following IRB approval. As part of standard procedure, the surgeon ligated blood supply to the diseased parathyroid gland prior to excision. Speckle contrast images were acquired before and less than one minute after the surgeon ligated the blood supply to the gland, in preparation for removal. To confirm removal of the hyperactive parathyroid gland, intraoperative measurement of PTH was performed before and 10 minutes after excision of the gland. Changes in speckle contrast between the vascularized and ligated state were assessed.

### Speckle contrast calculation

The number of pixels over which spatial speckle contrast is calculated is important, with too few pixels resulting in inaccuracy in contrast estimation while too many pixels sacrifice spatial resolution. Calculating speckle contrast over a 5 × 5 or 7 × 7 pixel region is generally considered a good compromise^30^. In this work, a 5 × 5 pixel window was used in calculating speckle contrast. The window is moved across the image of the acquired speckle pattern and at each location, the speckle contrast is calculated as the standard deviation of pixel intensity values within the window divided by the mean (Equation 1). The resultant speckle contrast image has values that theoretically range from 0 to 1, with values closer to 0 representing regions of greater vascular flow and values closer 1 representing regions of less vascular flow. It has been demonstrated that the quantity measured by LSCI is related to a product of speed and diameter of the vessel^31^. In this application, the microvasculature cannot be resolved due to the relatively large field of view required, and the measured speckle contrast is taken to be an ensemble measurement of the microvessels on the surface of the gland. Image acquisition and display is performed through a custom LabVIEW program, while a custom dynamic link library file enables processing and display at about 10 frames per second.

### Data analysis

Data was analyzed using MATLAB R2015a (The MathWorks Inc., Natick, MA). Speckle contrast images displayed in this manuscript are each an average of 20 consecutively generated maps. This was done to improve the spatial resolution of the final image. For any case where a sudden movement or a headlamp accidentally turned towards the surgical field caused a global sharp decrease in speckle contrast, these frames were excluded from averaged images. To correct for slight movement of the surgical field during recordings, an image registration algorithm was employed before averaging. The parathyroid gland was then demarcated using the “roipoly” function, avoiding bright spots caused by specular reflection, and the average speckle contrast within this region was calculated. In future iterations of the device, polarizers could be used to reduce specular reflection. The data was then grouped into vascularized and compromised according to the surgeon’s assessment of the gland. Compromised parathyroid glands were also further grouped into those that the surgeon decided to transplant and those that were left in place. Tests for statistical significance were performed using a two-sided two sample Student’s t-test and p-values less than 0.01 were considered statistically significant.

### Effect of ambient lighting on speckle contrast images

A microfluidic flow phantom made of PDMS and titanium dioxide to achieve a reduced scattering coefficient of 8 cm^−1^ was used to evaluate the effect of ambient lighting on speckle contrast images in the laboratory. The device had a 400 µm channel through which a solution of 0.4 g of milk powder diluted in 50 mL of water was flowed. Images were acquired with the room lights on and then off, and 20 speckle contrast images were averaged for each condition.

For one patient undergoing parathyroidectomy, two sets of measurements on one parathyroid gland were obtained to determine the effect of the OR lights on speckle contrast images. The surgeon first positioned the device so the parathyroid gland was in the field of view. Images were recorded with the operating room lights on, but with the surgical lights above the operating table pointing away from the surgical field. Then, without moving the device, all operating room lights were turned off and another set of images was obtained. For each condition, 20 speckle contrast images were averaged and the same region of interest was used to calculate average parathyroid gland speckle contrast.

### Data availability

The datasets generated and analysed during the current study are available from the corresponding author on reasonable request.
